# Comparing the efficacy and safety of thromboprophylaxis with enoxaparin *versus* normal saline after liver transplantation: randomized clinical trial

**DOI:** 10.1093/bjs/znae325

**Published:** 2025-02-24

**Authors:** Kunlin Xie, Hongzhao Yang, Shouping Wang, Chenghan Xiao, Tian Lan, Hanyu Jiang, Sheyu Li, Huakang Tu, Jian Yang, Tao Lyv, Jianguo Qiu, Jing Zhou, Zhongwei Zhang, Chengyou Du, Xifeng Wu, Jiwei Huang, Ahmed M Elgendi, Alfred W C Kow, Jiayin Yang, Yong Zeng, Hong Wu

**Affiliations:** Department of General Surgery, West China Hospital, Sichuan University, Chengdu, China; Liver Transplant Centre, Transplant Centre, West China Hospital, Sichuan University, Chengdu, China; Department of General Surgery, West China Hospital, Sichuan University, Chengdu, China; Liver Transplant Centre, Transplant Centre, West China Hospital, Sichuan University, Chengdu, China; Department of Critical Care Medicine, West China Hospital, Sichuan University, Chengdu, China; Department of Maternal and Child Health, West China School of Public Health and West China Fourth Hospital, Sichuan University, Chengdu, China; Institute of Systems Epidemiology, West China School of Public Health and West China Fourth Hospital, Sichuan University, Chengdu, China; Department of General Surgery, West China Hospital, Sichuan University, Chengdu, China; Liver Transplant Centre, Transplant Centre, West China Hospital, Sichuan University, Chengdu, China; Department of Radiology, West China Hospital, Sichuan University, Chengdu, China; Department of Endocrinology and Metabolism, West China Hospital, Sichuan University, Chengdu, China; Department of Guideline and Rapid Recommendation, Cochrane China Centre, MAGIC China Centre, Chinese Evidence-Based Medicine Centre, Chengdu, China; Department of Big Data in Health Science, Zhejiang University School of Public Health, Hangzhou, China; Centre of Clinical Big Data and Analytics of The Second Affiliated Hospital, Zhejiang University School of Medicine, Hangzhou, China; Department of General Surgery, West China Hospital, Sichuan University, Chengdu, China; Liver Transplant Centre, Transplant Centre, West China Hospital, Sichuan University, Chengdu, China; Department of General Surgery, West China Hospital, Sichuan University, Chengdu, China; Liver Transplant Centre, Transplant Centre, West China Hospital, Sichuan University, Chengdu, China; Department of Hepatobiliary Surgery, The First Affiliated Hospital of Chongqing Medical University, Chongqing, China; Department of Laboratory Medicine, West China Hospital, Sichuan University, Chengdu, China; Department of Critical Care Medicine, West China Hospital, Sichuan University, Chengdu, China; Department of Hepatobiliary Surgery, The First Affiliated Hospital of Chongqing Medical University, Chongqing, China; Department of Big Data in Health Science, Zhejiang University School of Public Health, Hangzhou, China; Division of Liver Surgery, Department of General Surgery, West China Hospital, Sichuan University, Chengdu, China; Department of Surgery, Faculty of Medicine, Alexandria University, Alexandria, Egypt; Division of HPB Surgery, Department of Surgery, National University of Singapore, Singapore; Department of General Surgery, West China Hospital, Sichuan University, Chengdu, China; Liver Transplant Centre, Transplant Centre, West China Hospital, Sichuan University, Chengdu, China; Division of Liver Surgery, Department of General Surgery, West China Hospital, Sichuan University, Chengdu, China; Department of General Surgery, West China Hospital, Sichuan University, Chengdu, China; Liver Transplant Centre, Transplant Centre, West China Hospital, Sichuan University, Chengdu, China

## Abstract

**Background:**

Venous thrombosis represents a significant complication after deceased-donor liver transplantation, yet there are currently no established protocols for thromboprophylaxis after deceased-donor liver transplantation. The aim of this study was to evaluate the efficacy and safety of prophylactic anticoagulation in patients undergoing deceased-donor liver transplantation.

**Methods:**

A dual-centre RCT of patients assigned to receive either enoxaparin or normal saline after liver transplantation was conducted. The primary efficacy outcome was the incidence of venous thrombosis (portal vein thrombosis and deep vein thrombosis) and the primary safety outcome was the incidence of major bleeding.

**Results:**

A total of 462 patients were recruited. In the intention-to-treat analysis, 89 patients (19.3%) experienced venous thrombosis and 141 patients (30.5%) experienced major bleeding within 90 days after transplantation. No significant differences were observed in the incidence of venous thrombosis, portal vein thrombosis, or deep vein thrombosis between the two groups in the intention-to-treat cohort. The anticoagulant group demonstrated a markedly elevated incidence of major bleeding (35.5% *versus* 25.5%, *P* = 0.020). Subgroup analysis revealed that anticoagulation was associated with a lower risk of deep vein thrombosis in hepatocellular carcinoma patients (HR 0.44 (95% c.i. 0.23 to 0.86), *P* = 0.016), without a significantly higher risk of major bleeding.

**Conclusion:**

Use of prophylactic anticoagulation with enoxaparin is associated with a significantly higher incidence of major bleeding in patients undergoing deceased-donor liver transplantation, rather than a lower likelihood of venous thrombosis.

**Registration number:**

ChiCTR2000032441 (www.chictr.org.cn).

## Teaser

Venous thrombosis represents a significant complication after deceased-donor liver transplantation, yet there are currently no established protocols for thromboprophylaxis after deceased-donor liver transplantation. This dual-centre RCT suggests that the administration of postoperative prophylactic anticoagulation to patients undergoing deceased-donor liver transplantation does not reduce the risks of venous thrombosis, but is associated with a higher incidence of major bleeding instead.

## Introduction

Vascular thrombosis poses a significant threat to patients undergoing liver transplantation and this issue has gained increasing importance given the growing numbers of donors and recipients with diabetes and obesity^1^. After liver transplantation, approximately 15.5% of recipients have reported experiencing deep vein thrombosis (DVT) with daily ultrasonography screening and upcoming complications such as pulmonary embolism (PE)^2,3^. Additionally, approximately 2% of patients have been diagnosed with hepatic artery thrombosis (HAT) and 3% of patients have been diagnosed with portal vein thrombosis (PVT) in the case of deceased donors^4,5^ and an even higher percentage of patients in the case of living donors or split donations^6^. The development of HAT and PVT can compromise blood supply to the transplanted liver and, in severe cases, result in elevated rates of graft loss, morbidity, and mortality for the recipient. The complications present a considerable challenge to the postoperative management plan.

Although thromboprophylaxis is well established as a means to reduce the risk of DVT after major abdominal and pelvic surgery^7,8^, it is unclear whether the same strategy will reduce the incidence of DVT or PVT among patients after liver transplantation^9–11^. In addition, it may increase the risk of post-liver transplantation morbidity and mortality due to haemorrhagic complications from the anticoagulation. As such, antithrombotic prophylaxis is not a standard practice after liver transplantation and, currently, there are no established protocols to guide the use of chemical thromboprophylaxis therapy in the post-transplant interval^12^. The need for further results from multicentre, prospective RCTs is evident to enhance thromboprophylaxis strategies after liver transplantation.

Enoxaparin, a low-molecular-weight heparin (LMWH) approved by the US Food and Drug Administration for the prevention of venous thromboembolism (VTE) at a daily dose of 40 mg, is frequently employed for postoperative chemical prophylaxis in patients undergoing orthopaedic, abdominal, and pelvic surgery^13–15^. Specifically, research has indicated that enoxaparin can effectively and safely prevent PVT in patients undergoing hepatectomy^16^.

The aim of this study was to investigate the efficacy and safety of chemical prophylaxis after liver transplantation. A prospective RCT was conducted to evaluate the relationship between daily enoxaparin administration in patients after liver transplantation and the occurrence of venous thrombosis and major bleeding events.

## Methods

### Trial design, study participants, and study ethics

This was a prospective RCT aimed at evaluating the roles of chemical thromboprophylaxis after deceased-donor liver transplantation (DDLT). This study received approval from the Institutional Review Board of West China Hospital, Sichuan University, and was registered in the Chinese Clinical Trial Registry (ChiCTR2000032441) before patient enrolment. All liver transplantations conducted between April 2020 and August 2023 at two high-volume liver transplantation centres in China (West China Hospital, Sichuan University, and the First Affiliated Hospital of Chongqing Medical University) were included in eligibility assessment and patients who provided informed consent before liver transplantation were included in this study. This study was conducted in compliance with Good Clinical Practice guidelines and the Declaration of Helsinki.

The authors declare that no organs were procured from executed prisoners and that organs were procured after informed consent or authorization.

### Inclusion criteria

The study included all orthotopic DDLT patients aged between 18 and 70 years. The exclusion criteria included: living-donor liver transplantation, split liver transplantation, or autotransplantation; and other ongoing anticoagulative therapy. The exclusion criteria for the per-protocol (PP) analysis included: patients in the anticoagulant group who never received enoxaparin because of severe coagulation disorders (defined as a platelet count less than 30 000/μl); patients for whom any endpoint events (venous thrombosis, major bleeding, or death) occurred before the first dose of enoxaparin or normal saline; and patients who started renal replacement therapy (RRT) within the first 7 days after liver transplantation.

All transplantations were performed in collaboration with senior surgeons and dedicated anaesthesia teams. The position of surgeon-in-chief was filled by a senior physician with over 10 years of experience in liver surgery and a track record of performing more than 30 liver transplants annually over the past 3 years. Regarding the inferior vena cava (IVC) anastomosis technique, the piggyback approach has primarily been used at the research centres since August 2022. Previously, most patients underwent IVC anastomosis using the cavaplasty technique^17^. No other alterations were made to the surgical techniques employed in DDLT operations during the study interval.

### Randomization and intervention

Patients were randomly allocated (1 : 1) to either the anticoagulant group or the control group using stratified randomization, with the allocation stratified according to six different surgeons. Randomization was performed using SPSS^®^ (IBM, Armonk, NY, USA; Statistics 26.0). A computer-generated random number list was used for group allocation, with subject numbers placed in sealed, unmarked envelopes. The envelopes were opened to determine group assignments for each subject. Patient recruitment and the informed consent process were carried out by the surgeons, whereas randomization was performed by an independent statistician who was not involved in participant recruitment or the study intervention. The group allocation was concealed from the patients and physicians in the ICU where the patients would be treated for about 1 week after liver transplantation. However, the surgeon was aware of group allocation and was responsible for prescribing the interventions and related tests (for example anti-Xa levels). Patients in the anticoagulant group received a daily dose of 40 mg enoxaparin within 24 h after their surgery and continued this regimen until day 7 after liver transplantation, provided that the platelet count was greater than or equal to 30 000/μl, the estimated glomerular filtration rate (eGFR) was greater than 30 ml/min/1.73 m^2^, and there were no major bleeding events. To assess the efficacy of prophylactic anticoagulation, anti-Xa levels were measured 4 h after the third dose of enoxaparin. To ensure compliance with the protocol, surgeons, ICU physicians, and nurses were trained in the use and monitoring of enoxaparin. The control group received a daily dose of normal saline (0.9% NaCl) within 24 h after their surgery, with this regimen continuing until day 7 after liver transplantation. All medication and monitoring information was recorded in an electronic medical order form in the Hospital Information System for review.

### Outcomes and data collection

The primary efficacy outcome was the incidence of venous thrombosis events within the first 90 days after liver transplantation. Venous thrombosis events included all the PVT and DVT events in this study. The primary safety outcome was defined as major bleeding events within the first 90 days after liver transplantation according to the International Society on Thrombosis and Haemostasis (ISTH) criteria^18^: bleeding leading to a haemoglobin (Hb) level decrease of 1.24 mmol/l (20 g/l) or more, or necessitating a transfusion of 2 units or more of whole blood or packed red blood cells (RBCs) within 24 h. The secondary efficacy outcomes included the all-cause mortality and the incidence of PVT and DVT events within the first 90 days after liver transplantation.

Patients with intraoperative blood loss greater than 2000 ml or a platelet count less than 30 000/μl for more than 4 days within 7 days after surgery were considered at high risk of postoperative bleeding. Similarly, those with a history of preoperative variceal bleeding, preoperative PVT, portal vein thrombectomy, or non-physiological reconstruction of portal vein inflow were deemed at high risk of postoperative PVT according to the literature^12^.

All patients were subsequently transferred to the ICU after liver transplantation. Doppler ultrasonography (DUS) was conducted by ultrasonography physicians who were unaware of the intervention that patients had received daily during the initial 2-week interval, on a weekly basis during the third and fourth postoperative weeks and on a biweekly basis during the second and third months after liver transplantation. This was done to confirm the patency of the portal vein and extremity veins. No postoperative physiotherapy was implemented. The diagnosis of PVT and DVT after liver transplantation was based on the results of DUS findings. Once a diagnosis of PVT or DVT was established, therapeutic anticoagulation with LMWH and rivaroxaban was initiated for a minimum of 6 months. A subset of patients with PVT underwent surgical or percutaneous thrombectomy. All patients underwent plain chest CT within 1 week after surgery to detect any lung abnormalities. For those diagnosed with DVT via DUS, chest CT angiography (CTA) was performed to screen for PE if their PaO₂/FiO₂ ratio (the ratio of arterial oxygen partial pressure to inspired oxygen fraction) dropped below 200, with cases of pleural effusion and pulmonary atelectasis excluded. The treatment of postoperative bleeding included an RBC transfusion, which was carried out in accordance with the National Institute for Health and Care Excellence (NICE) blood transfusion guidelines^19^. The amount of RBCs to be transfused was calculated based on the properties and volume of drainage fluid and the pre-transfusion Hb levels. In the absence of active bleeding, only 1 unit of RBCs was transfused in a single session. In the event of suspected active abdominal haemorrhage that could not be controlled by blood transfusion and conservative treatment, surgical intervention was promptly performed. Fatal bleeding was defined as postoperative haemorrhage that directly resulted in a patient’s death.

After surgery, routine blood tests, liver and kidney function assessments, rapid thrombelastogram (rTEG), and monitoring of protein S (PS), protein C (PC), and anti-Xa levels were conducted. Maintenance immunosuppression was achieved through the administration of tacrolimus, mycophenolate, and prednisone. Calcineurin inhibitor therapy was initiated on the first or second postoperative day and standard recipient variables and patient histories were recorded.

### Statistical analysis

The aim of this study was to determine whether there was a statistically significant difference in the incidence of thrombosis events between the anticoagulant and control groups. The study centres employed a more rigorous thrombus screening strategy in post-transplant patients, comprising daily DUS screening. This strategy enabled the detection of a significant number of asymptomatic isolated calf muscle vein thrombosis (ICMVT) cases, as observed in the experience of study centres. A previous study reported a 15.5% incidence of DVT in patients who were screened daily using DUS^2^, which is similar to the incidence previously experienced by the authors. It was therefore hypothesized that the incidence of venous thrombosis events in the control group would be 18% (DVT, 15%; and PVT, 3%). In light of the proven efficacy of enoxaparin in patients undergoing major orthopaedic surgery and general surgery, the authors postulated that enoxaparin would result in a 50% reduction in venous thrombosis events (18% to 9% in the anticoagulant group)^20–22^. Furthermore, a 2.5% dropout rate within 90 days after surgery was assumed, given the high compliance of post-liver transplantation patients and the relatively short follow-up interval for the primary outcomes. Considering the assumptions, the sample size was determined to be 229 patients in each group. Further details can be found in the study protocol (*Supplementary Methods*).

For the description of the sample, continuous variables that exhibited a normal distribution are presented as mean(s.d.), whereas those that did not conform to a normal distribution are presented as median (interquartile range (i.q.r.)). Categorical variables are presented as *n* (%).

Both intention-to-treat (ITT) and PP analyses were conducted for all outcomes. All allocated patients were included in the ITT population, regardless of compliance. In contrast, the PP population comprised only those who received the intervention according to the protocol, excluding patients with protocol violations as defined by the PP analysis exclusion criteria. To ascertain the dissimilarities in the primary outcome, the Pearson chi-squared test was employed and risk ratios (RRs) and their 95% confidence intervals were calculated. The Kaplan–Meier method was used to estimate survival probabilities, with log rank *P* values for comparisons. The cumulative incidence function (CIF) was generated for thrombosis (venous thrombosis, PVT, and DVT) and major bleeding events, with comparisons evaluated using Gray’s test^23^. For competing risk analyses, thrombosis, major bleeding, and all-cause death events were treated as competing events relative to each other. Cox proportional hazards models were applied to estimate HRs for survival outcomes and Fine-Gray subdistribution hazard models were used to calculate HRs for thrombosis and major bleeding events^24^. The selection of potential confounding variables for adjustment was informed by literature review, with details provided in the *Supplementary Methods*. Both adjusted and unadjusted HRs are reported with 95% confidence intervals. Exploratory subgroup analyses of primary and secondary outcomes were conducted for predefined subgroups, including patients with hepatocellular carcinoma (HCC), Model for End-Stage Liver Disease (MELD) scores greater than or equal to 30, a high risk of postoperative bleeding, and a high risk of postoperative PVT. HRs with 95% confidence intervals for these subgroups were estimated and are presented as forest plots for exploratory purposes. Missing data were imputed using the median values (applies to operating time and intraoperative blood loss). Statistical significance was defined as a two-sided *P* value of less than 0.050. Further details can be found in the *Supplementary Methods*.

All statistical analyses were conducted using SPSS^®^ (IBM, Armonk, NY, USA; Statistics 26.0) and R version 4.4.0 (R Foundation for Statistical Computing, Vienna, Austria).

## Results

### Patient characteristics

**Fig. 1:**
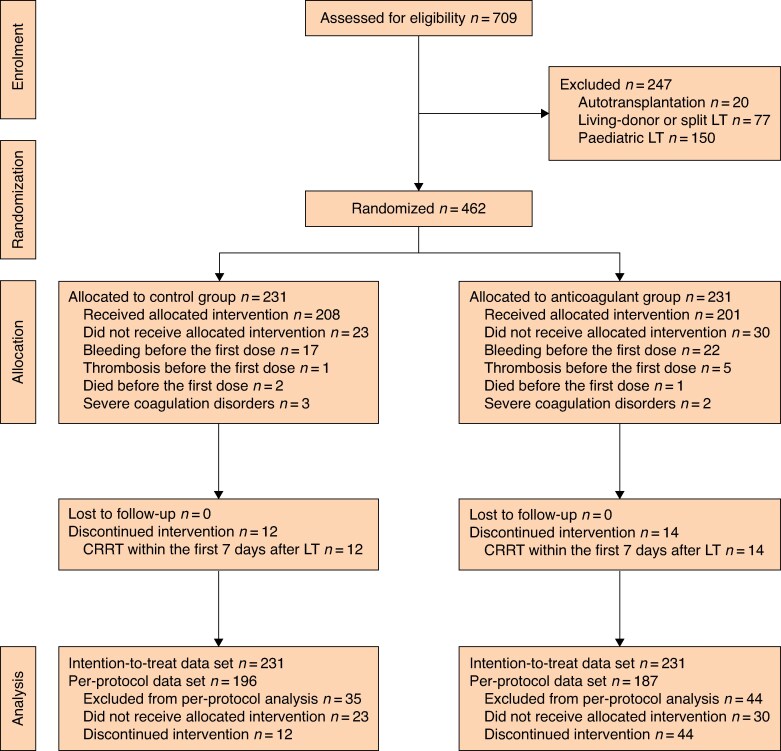
CONSORT diagram for trial LT, liver transplantation; CRRT, continuous renal replacement therapy.

A total of 709 liver transplantation patients were screened for eligibility between 13 April 2020 and 9 August 2023. Excluding patients who underwent autotransplantation (20 patients), living-donor or split liver transplantation (77 patients), and paediatric liver transplantation (150 patients), 462 patients were randomized and included in the ITT analysis. After the exclusion of patients who did not adhere to the protocol because of severe coagulation disorders (defined as a platelet count less than 30 000/μl), and those who experienced endpoint events before the first dose or underwent RRT within the first 7 days after liver transplantation, 383 patients were included in the PP analysis. Among the patients, 187 received postoperative prophylactic anticoagulation, as illustrated in *Fig. 1*. Due to the high level of compliance among liver transplantation recipients and the relatively brief 90-day follow-up interval for the primary outcome, no patients were lost to follow-up for the primary outcomes in either group. The median follow-up interval for overall survival was 19.3 months, with a range of 1 to 50 months. Regarding the patients who were randomly assigned, the median age was 51 years, 369 were male and 93 were female, and the median MELD score was 16 (i.q.r. 10–23) (*Table 1*).

**Table 1:** Clinical characteristics at baseline in the intention-to-treat population

	Total (*n* = 462)	Anticoagulant (*n* = 231)	Control (*n* = 231)
**Sex**			
Male	369 (79.9)	183 (79.2)	186 (80.5)
Female	93 (20.1)	48 (20.8)	45 (19.5)
Age (years), median (i.q.r.)	51.00 (44.00–56.00)	52.00 (44.00–57.00)	51.00 (43.00–56.00)
BMI (kg/m^2^), mean(s.d.)	23.66 (3.34)	23.53 (3.36)	23.79 (3.32)
Chronic viral hepatitis	349 (75.5)	173 (74.9)	176 (76.2)
HCC	215 (46.5)	114 (49.4)	101 (43.7)
Hepatic encephalopathy	48 (10.4)	20 (8.7)	28 (12.1)
Diabetes	76 (16.5)	42 (18.2)	34 (14.7)
Hypertension	46 (10.0)	21 (9.1)	25 (10.8)
**Preoperative PVT**	53 (12.6)	27 (13.9)	26 (11.3)
Yerdel grade I	19 (35.8)	8 (29.6)	11 (42.3)
Yerdel grade II	30 (56.6)	16 (59.3)	14 (53.8)
Yerdel grade III	2 (3.8)	2 (7.4)	0 (0.0)
Yerdel grade IV	2 (3.8)	1 (3.7)	1 (3.8)
Preoperative TIPS	40 (8.7)	21 (9.1)	19 (8.2)
Preoperative MELD score, median (i.q.r.)	16 (10–23)	15 (10–23)	16 (11–23)
Preoperative Hb (g/l), median (i.q.r.)	107.00 (88.00–131.00)	109.00 (86.50–132.00)	105.00 (88.00–130.50)
Preoperative platelet count (10^9^/l), median (i.q.r.),	61.00 (38.00–95.00)	56.50 (36.00–92.75)	65.00 (41.00–97.00)
Operating time (h), median (i.q.r.)	6.50 (5.50, 7.84)	6.60 (5.71, 7.74)	6.50 (5.32, 7.95)
**IVC anastomosis technique**			
Cavaplasty	318 (68.8)	167 (72.3)	151 (65.4)
Piggyback	144 (31.2)	64 (27.7)	80 (34.6)
Intraoperative blood loss (ml), median (i.q.r.)	1200.00 (800.00–2000.00)	1200.00 (800.00–2000.00)	1000.00 (800.00–2000.00)
Intraoperative RBC transfusion (units), median (i.q.r.)	6.00 (2.00–10.00)	6.00 (2.00–10.00)	6.00 (3.00–10.00)
Intraoperative plasma transfusion (ml), median (i.q.r.)	750.00 (200.00–1350.00)	775.00 (350.00–1387.50)	600.00 (187.50–1300.00)

Values are *n* (%) unless otherwise indicated. i.q.r., interquartile range; HCC, hepatocellular carcinoma; PVT, portal vein thrombosis; TIPS, transjugular intrahepatic portosystemic shunt; MELD, model of end stage liver disease; Hb, haemoglobin; IVC, inferior vena cava; RBC, red blood cell.

In the anticoagulation group, 49 patients were monitored for anti-Xa concentration to evaluate the efficacy of anticoagulation. The mean(s.d.) anti-Xa concentration was 0.45(0.24) IU/ml, with 33 out of 49 patients (67.3%) falling within the target range of 0.20–0.54 IU/ml. The number of patients with anti-Xa levels less than 0.2 IU/ml was 3 out of 49 (6.1%) and the number of patients with anti-Xa levels greater than 0.54 IU/ml was 13 out of 49 (26.5%) (*Fig. S1*).

### Efficacy outcomes

**Fig. 2:**
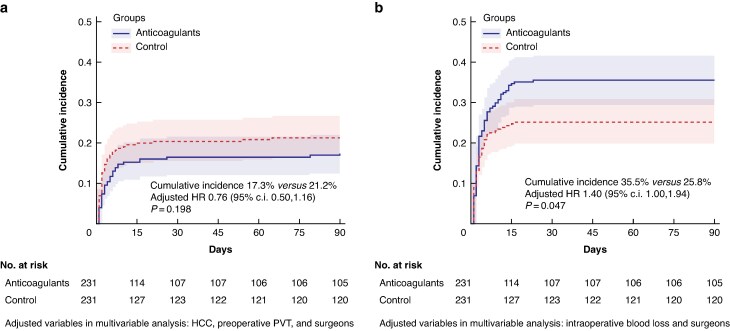
Competing risk of primary outcomes **a** Competing risk of postoperative venous thrombosis event. **b** Competing risk of postoperative major bleeding event. HCC, hepatocellular carcinoma; PVT, portal vein thrombosis.

In the ITT population, a total of 40 patients (17.3%) in the anticoagulant group and 49 patients (21.2%) in the control group experienced venous thrombosis events within the first 90 days after liver transplantation, with no significant difference observed between the groups (RR 0.82 (95% c.i. 0.56 to 1.19), *P* = 0.288). The results of the PP analysis were comparable (*Table 2*). The cumulative incidence curves demonstrated comparable outcomes (*Fig. 2a*). After the adjustment for potential confounding variables (illustrated in *Fig. 2a*), the HR for venous thrombosis events was 0.76 (95% c.i. 0.50–1.16) (*P* = 0.198). Unadjusted HRs are reported in *Table S1*.

**Table 2:** Efficacy outcomes in the intention-to-treat and per-protocol populations

	Total	Anticoagulant	Control	Risk ratio (95% c.i.)	*P*
**Intention to treat**	462 (100.0)	231 (100.0)	231 (100.0)		
Venous thrombosis within the first 90 days after LT	89 (19.3)	40 (17.3)	49 (21.2)	0.82 (0.56,1.19)	0.288
PVT within the first 90 days after LT	15 (3.2)	8 (3.5)	7 (3.0)	1.14 (0.42,3.10)	0.793
DVT within the first 90 days after LT	73 (15.8)	31 (13.4)	42 (18.2)	0.74 (0.48,1.13)	0.161
ICMVT	57 (12.3)	22 (9.5)	35 (15.2)	0.63 (0.38,1.04)	0.066
PDVT	16 (3.5)	9 (3.9)	7 (3.0)	1.29 (0.49,3.39)	0.611
All-cause mortality	63 (13.6)	31 (13.4)	32 (13.9)	0.97 (0.61,1.53)	0.892
Perioperative all-cause mortality*	44 (9.5)	22 (9.5)	22 (9.5)	1.00 (0.57,1.75)	1.000
**Per protocol, as treated**	383 (100.0)	187 (100.0)	196 (100.0)		
Venous thrombosis within the first 90 days after LT	74 (19.3)	29 (15.5)	45 (23.0)	0.67 (0.44,1.03)	0.065
PVT within the first 90 days after LT	12 (3.1)	6 (3.2)	6 (3.1)	1.05 (0.34,3.19)	0.934
DVT within the first 90 days after LT	61 (15.9)	22 (11.8)	39 (19.9)	0.59 (0.37,0.96)	0.030†
ICMVT	47 (12.3)	14 (7.5)	33 (16.8)	0.45 (0.25,0.80)	0.005†
PDVT	14 (3.7)	8 (4.3)	6 (3.1)	1.40 (0.49,3.95)	0.526
All-cause mortality	37 (9.7)	18 (9.6)	19 (9.7)	0.99 (0.54,1.83)	0.982
Perioperative all-cause mortality*	22 (5.7)	11 (5.9)	11 (5.6)	1.05 (0.47,2.36)	0.910

Values are *n* (%) unless otherwise indicated. *Death occurred within the first 30 days after liver transplantation. †Statistically significant. LT, liver transplantation; PVT, portal vein thrombosis; DVT, deep vein thrombosis; ICMVT, isolated calf muscle vein thrombosis; PDVT, proximal deep vein thrombosis.

The incidence of all-cause mortality was 31 (13.4%) in the anticoagulant group and 32 (13.9%) in the control group. Of these deaths, 22 (9.5%) occurred perioperatively within 30 days of surgery in both groups (*Table 2*). The most common causes of postoperative death were infection, followed by graft failure, PVT or HAT, and major bleeding. The 1-year post-transplant overall survival was 87.3% in the anticoagulant group and 86.9% in the control group and the 3-year overall survival was 86.1% in the anticoagulant group and 85.3% in the control group (*Table S2* and *Fig. S2e*,*f*).

A total of eight patients (3.5%) in the anticoagulant group and seven patients (3.0%) in the control group exhibited PVT events within the first 90 days after liver transplantation (*Table 2* and *Fig. S2a*). A total of 31 patients (13.4%) in the anticoagulant group and 42 patients (18.2%) in the control group were noted to have experienced DVT events within the first 90 days after liver transplantation (*Table 2* and *Fig. S2b*). It is noteworthy that a more rigorous screening strategy for DVT detected more asymptomatic ICMVT at an earlier stage in the postoperative interval, rather than after they progressed to symptomatic proximal DVT (PDVT). In the PP population, prophylactic anticoagulation significantly reduced the incidence of ICMVT (7.5% *versus* 16.8%, *P* = 0.005), with an adjusted HR of 0.58 (95% c.i. 0.34–1.00) (*P* = 0.049) in the ITT population. In contrast, no statistically significant difference in PDVT was observed between the two groups (*Table 2* and *Fig. S2c*,*d*). The results of the other efficacy outcomes in the PP population were comparable to those of the ITT population (*Table 2*). Among patients who developed a postoperative DVT, there were no instances of PE observed in either group.

### Safety outcomes

In the ITT population, major bleeding events occurred within the first 90 days after liver transplantation in 82 patients (35.5%) in the anticoagulant group and in 59 patients (25.5%) in the control group (RR 1.39 (95% c.i. 1.05 to 1.84), *P* = 0.020) (*Table 3*). Furthermore, the cumulative incidence curves indicated that the cumulative incidence of major bleeding was significantly higher in the anticoagulation group (*Fig. 2b*). After adjusting for potential confounding factors (illustrated in *Fig. 2b*), the HR for major bleeding events was 1.40 (95% c.i. 1.00 to 1.94) (*P* = 0.047). Unadjusted HRs are reported in *Table S1*. The results of the PP population were comparable (*Table 3*).

**Table 3:** Safety outcomes in the intention-to-treat and per-protocol populations

	Total	Anticoagulant	Control	Risk ratio (95% c.i.)	*P*
**Intention to treat**	462 (100.0)	231 (100.0)	231 (100.0)		
Major bleeding in the first 90 days after LT	141 (30.5)	82 (35.5)	59 (25.5)	1.39 (1.05,1.84)	0.020*
Decrease in Hb level ≥20 g/l†	121 (26.2)	70 (30.3)	51 (22.1)	1.37 (1.01,1.87)	0.044*
RBC transfusion ≥2 units caused by bleeding†	32 (6.9)	21 (9.1)	11 (4.8)	1.90 (0.94,3.87)	0.067
Intracranial haemorrhage	2 (0.4)	1 (0.4)	1 (0.4)	1.00 (0.06,15.89)	1.000
Fatal bleeding	4 (0.9)	1 (0.4)	3 (1.3)	0.33 (0.03,3.18)	0.616
Surgical interventions	8 (1.7)	3 (1.3)	5 (2.2)	0.60 (0.15,2.48)	0.476
**Per protocol, as treated**	383 (100.0)	187 (100.0)	196 (100.0)		
Major bleeding in the first 90 days after LT	85 (22.2)	54 (28.9)	31 (15.8)	1.83 (1.23,2.71)	0.002*
Decrease in Hb level ≥20 g/l†	74 (19.3)	48 (25.7)	26 (13.3)	1.93 (1.25,2.98)	0.002*
RBC transfusion ≥2 units caused by bleeding†	19 (5.0)	14 (7.5)	5 (2.6)	2.93 (1.08,7.99)	0.026*
Intracranial haemorrhage	1 (0.3)	0 (0.0)	1 (0.5)	–	1.000
Fatal bleeding	1 (0.3)	1 (0.5)	0 (0.0)	–	0.488
Surgical interventions	4 (1.0)	1 (0.5)	3 (1.5)	0.35 (0.04,3.33)	0.649

LT, liver transplantation; Hb, haemoglobin; RBC, red blood cell. *Statistically significant. †Within 24 h.

The median time to major bleeding was 1 day after the final intervention dose in both groups. Of those who experienced a major bleeding, 45 (83.5%) in the anticoagulant group and 27 (87.0%) in the control group experienced a major bleeding within 2 days of the last intervention dose. A total of 21 patients (9.1%) in the anticoagulant group and 11 patients (4.8%) in the control group received an RBC transfusion of greater than or equal to 2 units within 24 h due to major bleeding. A total of three patients (1.3%) in the anticoagulant group and five patients (2.2%) in the control group underwent surgical interventions. One patient (0.4%) in the anticoagulant group and three patients (1.3%) in the control group experienced fatal bleeding. Intracranial haemorrhage was observed in one patient (0.4%) in each group (*Table 3*). Moreover, no cases of heparin-induced thrombocytopenia were observed in patients who received prophylactic anticoagulation.

### Exploratory subgroup analysis

**Fig. 3:**
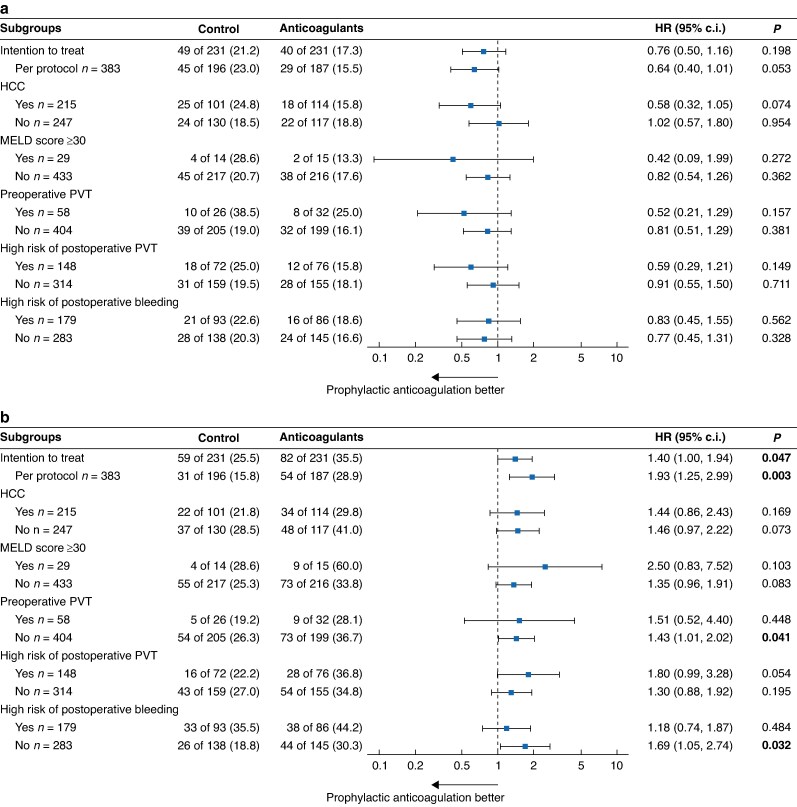
Exploratory subgroup analysis: HRs of primary outcomes in subgroups **a** HRs of postoperative venous thrombosis event in subgroups. **b** HRs of postoperative major bleeding event in subgroups. HCC, hepatocellular carcinoma; MELD, Model for End-Stage Liver Disease; PVT, portal vein thrombosis.

The subgroup analysis revealed no statistically significant difference between the prophylactic anticoagulation and control groups in terms of the risk of venous thrombosis (*Fig. 3a*). Conversely, the risk of major bleeding was significantly elevated in several subgroups, including those patients who were not at a high risk of bleeding (*Fig. 3b*). A trend towards an increased risk of major bleeding in the anticoagulation group was observed in most subgroups, although the difference did not reach statistical significance.

The results demonstrated that prophylactic anticoagulation did not result in a reduction in the risk of PVT in the subgroups of preoperative PVT and high-risk postoperative PVT (*Fig. S3a*). Although prophylactic anticoagulation did not reduce the risk of DVT across most subgroups, a lower risk of DVT was observed in patients with HCC who received prophylactic anticoagulation (HR 0.44 (95% c.i. 0.23 to 0.86), *P* = 0.016) (*Fig. S3b*). Notwithstanding the elevated risk of major bleeding, prophylactic anticoagulation did not influence the prognosis of patients (*Fig. S3c*).

## Discussion

In this study, prophylactic anticoagulation with enoxaparin in liver transplantation recipients did not reduce the incidence of postoperative PVT and DVT, even in patients with high-risk factors. Instead, it increased the risk of bleeding. As far as the authors know, this is the only prospective RCT looking at prophylactic anticoagulation after liver transplantation.

The results of the present study challenge the practice of administering routine prophylactic anticoagulation after liver transplantation, as it did not provide any clear benefits in terms of reducing postoperative thrombosis and may even have harmful effects. These findings diverge from the approach of institutions that routinely employ prophylactic anticoagulation after liver transplantation^25,26^. There is limited retrospective research on the effectiveness of prophylactic anticoagulation in reducing thrombosis risk after liver transplantation and the existing studies have yielded inconsistent outcomes^10^. Although the consensus of the Spanish Society of Liver Transplantation and the Spanish Society of Thrombosis and Haemostasis recommends postoperative anticoagulation in high-risk patients^12^, a retrospective study found that administering therapeutic doses of anticoagulation after surgery did not decrease the frequency of thrombosis in patients with preoperative Yerdel grade I/II PVT^27^. Expert consensus does not provide clear directives on the necessity of anticoagulation in patients lacking risk factors for vascular thrombosis. The present study suggests that prophylactic anticoagulation with enoxaparin after liver transplantation does not lower the incidence of PVT and DVT. This is consistent with a recent meta-analysis that concluded prophylactic anticoagulation offers no clear benefits to adult liver transplantation recipients^12,28^.

In the present study, the rate of PVT was 3.2%, in line with prior literature. In recent years, the incidence of HAT and PVT has generally decreased due to advancements in microsurgical techniques and perioperative management^29^. Although the present study indicates that enoxaparin is not an effective prophylactic anticoagulation agent after liver transplantation, the use of certain medications such as aspirin or statins may reduce the occurrence of vascular complications (for example arterial thrombosis) after the procedure^30–32^.

Although there was no significant difference in venous thrombosis between the two groups, the anticoagulation group exhibited a significantly decreased risk of ICMVT in the ITT and PP analysis. Nevertheless, this did not result in a reduced risk of PDVT for patients receiving anticoagulation. It was postulated that the lack of an increase in PDVT in the control group was because ultrasonography was employed to screen for ICMVT, after which therapeutic anticoagulation was initiated. This may have prevented the further progression of asymptomatic ICMVT to symptomatic PDVT. Consequently, the preventive role of prophylactic anticoagulation for ICMVT may have been superseded by the strategy of ultrasonography screening, given the possible increased risk of bleeding associated with prophylactic anticoagulation.

In terms of safety, the present study identified an increased risk of major bleeding in the prophylactic anticoagulation group, a finding that remained consistent even after accounting for potential confounders and conducting subgroup analyses. Despite the general acceptance of 40 mg enoxaparin once daily as safe for prophylactic anticoagulation after surgery, this may not hold true for liver transplantation patients. End-stage liver disease is characterized by a rebalancing of haemostasis achieved through a decrease in both pro- and anti-haemostatic elements. As a surgical treatment for end-stage liver disease, liver transplantation has an additional impact on a patient’s coagulation function, increasing the risk of perioperative major bleeding and vascular complications. During the initial postoperative interval after liver transplantation, the delicate balance of a patient’s coagulation function can easily tip towards either bleeding or thrombotic complications. Earlier retrospective studies have also indicated an increased bleeding risk associated with anticoagulants^28,33^. Cases of cerebral haemorrhage after liver transplantation have been reported and it has been suggested that postoperative anticoagulation may contribute to the development of cerebral haemorrhage in instances of cerebral ischaemia due to perioperative haemodynamic instability^34^.

The findings of the present study suggest that enoxaparin is effective in preventing DVT in HCC patients without increasing the risk of major bleeding. This can be explained by the fact that HCC patients are at a high risk of DVT due to their malignancy, age, and prolonged operating time, making them likely to benefit from anticoagulation. On the flip side, patients who undergo liver transplantation for HCC typically exhibit stable coagulation function during the perioperative interval. In fact, liver transplantation procedures in the present study are not significantly different from regular liver surgeries in terms of duration or bleeding risk, and, as a result, the patients in the present study are less susceptible to adverse effects associated with anticoagulation.

Nevertheless, the present study has several limitations. The use of a 40-mg once-daily dose of enoxaparin for 1 week as a prophylactic anticoagulation regimen for liver transplantation may differ from practices at other centres. However, this regimen represents a classic approach to prophylactic anticoagulation and the present study provides clinical justification for its use. Second, the limited number of thrombotic events in the present study may have resulted in insufficient statistical power; in particular, the number of PVT events that occurred was too low because of the inherently low incidence of PVT, which could have led to a false-negative result. The authors can only conclude that their results did not support the hypothesis that prophylactic anticoagulation with enoxaparin would reduce venous thrombosis events by 50%. Third, as the authors’ diagnostic strategy for PE does not entail screening all patients with DVT using CTA, the authors recognize that the possibility of PE cannot be entirely discounted, particularly in patients with subclinical symptoms or in cases where PE may have been masked by other postoperative respiratory disturbances. Furthermore, the male : female sex ratio in the present study (approximately 8 : 2) is slightly higher than that observed in other current clinical studies of liver transplantation worldwide (approximately 7 : 3)^35,36^. As a result, the generalizability of the findings of the present study may be limited. Finally, the conclusions drawn from the present study pertain specifically to cadaveric liver transplantation, excluding those who underwent paediatric, living-donor, and split liver transplantation. Therefore, the efficacy of prophylactic anticoagulation in these specific patient groups should be confirmed through appropriate prospective randomized controlled studies.

## Supplementary Material

znae325_Supplementary_Data

## Data Availability

The data that support the findings of this study are available from the corresponding author upon reasonable request.
