# Supplementary material for: Comparing the efficacy and safety of thromboprophylaxis with enoxaparin versus normal saline after liver transplantation: randomized clinical trial
Source: Br J Surg. 2025 Feb 24;112(2):znae325. doi: 10.1093/bjs/znae325 (PMC11848516; doi:10.1093/bjs/znae325)
Supplement: znae325_Supplementary_Data [file znae325_supplementary_data.zip › Supplementary_Material.docx]

**Randomized controlled trial comparing efficacy and safety of thromboprophylaxis with enoxaparin vrs normal saline after liver transplantation**

Kunlin Xie^1,2^, Hongzhao Yang^1,2^, Shouping Wang^3^, Chenghan Xiao^4,5^, Tian Lan^1,2^, Hanyu Jiang^6^, Sheyu Li^7^, Huakang Tu^8^, Jian Yang^1,2^, Tao Lyv^1,2^, Jianguo Qiu^9^, Jing Zhou^10^, Zhongwei Zhang^11^, Chengyou Du^9^, Xifeng Wu^8^, Jiwei Huang^12^, Ahmed Mohamed Elgendi^13^, Alfred Wei-Chieh Kow^14^, Jiayin Yang^1,2^, Yong Zeng^12^, Hong Wu^1,2^

1. Department of General Surgery, West China Hospital, Sichuan University, Chengdu, China.

2. Liver Transplant Center, Transplant Center, West China Hospital, Sichuan University, Chengdu, China.

3. Department of Critical Care Medicine, West China Hospital, Sichuan University, Chengdu, China.

4. Department of Maternal and Child Health, West China School of Public Health and West China Fourth Hospital, Sichuan University.

5. Institute of Systems Epidemiology, West China School of Public Health and West China Fourth Hospital, Sichuan University, Chengdu, China

6. Department of Radiology, West China Hospital, Sichuan University, Chengdu, China.

7. Department of Endocrinology and Metabolism and Department of Guideline and Rapid Recommendation, Cochrane China Center, MAGIC China Center, Chinese Evidence-Based Medicine Center, West China Hospital, Sichuan University, Chengdu, China.

8. Department of Big Data in Health Science, School of Public Health, Center of Clinical Big Data and Analytics of The Second Affiliated Hospital, Zhejiang University School of Medicine, Hangzhou, Zhejiang, China.

9. Department of Hepatobiliary Surgery, The First Affiliated Hospital of Chongqing Medical University, Chongqing, China.

10. Department of Laboratory, West China Hospital, Sichuan University, Chengdu, China.

11. Department of Critical Care Medicine, West China Hospital, Sichuan University, Chengdu, China.

12. Division of Liver Surgery, Department of General Surgery, West China Hospital, Sichuan University, Chengdu, China.

13. Department of Surgery, Faculty of Medicine, Alexandria University, Egypt

14. Division of HPB Surgery, Department of Surgery, National University of Singapore, Singapore, Singapore.

**Corresponding authors**

Dr. Hong Wu, 37 Guoxue Alley, Chengdu, China.

**Supplementary Materials - Index**

| **Supplementary Methods** |  |
| --- | --- |
| Trial Design, Settings & Eligibility Criteria | *Pag. 3* |
| Interventions | *Pag. 3* |
| Clinical Outcomes | *Pag. 4* |
| Sample Size & Randomization | *Pag. 5* |
| Statistical Analysis | *Pag. 6* |
| Ethics & Others | *Pag. 8* |
| Changes to Protocol after Trial Commencement | *Pag. 9* |
| **Supplementary Results** |  |
| HAT | *Pag. 10* |
| Laboratory indicators within 7 days postoperatively | *Pag. 10* |
| **Supplementary Figures and Tables** |  |
| Figure S1 | *Pag. 11* |
| Figure S2 | *Pag. 11* |
| Figure S3 | *Pag. 12* |
| Figure S4 | *Pag. 13* |
| Table S1 | *Pag. 13* |
| Table S2 | *Pag. 13* |
| **References** | *Pag. 14* |

Supplementary Methods

1. **Trial Design, Settings & Eligibility Criteria**
   1. Trial Design

A Dual-Center, parallel, prospective randomized controlled trial.

- 1. Institute’s name and address
     1. West China Hospital, Sichuan University, 37 Guoxue Alley, Chengdu, China.
     2. The First Affiliated Hospital of Chongqing Medical University, No.1 Youyi Road, Chongqing, China.
  2. Inclusion Criteria
     1. All orthotopic deceased-donor liver transplantation patients aged between 18 and 70 years.
     2. Signed informed consent and able to comply with the requirements of the program; if the patient is unable to sign the informed consent form, his/her legal guardian or representative must sign it.
  3. Exclusion Criteria
     1. Autotransplantation, living donor, split and pediatric liver transplantation.
     2. Other ongoing anticoagulative therapy.
  4. Exclude Criteria for Per-protocol Analysis
     1. Patients in the anticoagulants group who never received intervention because of severe coagulation disorders.
     2. Patients were found that any end point events (VT, major bleeding or death) before the first dose of intervention.
     3. Patients who started renal replacement therapy (RRT) within first 7 days post-LT.
     4. Patients are allergic to enoxaparin or develop heparin induced thrombocytopenia.
     5. Subjects or their legal representatives requested to withdraw from the study.

1. **Interventions**
   1. Anticoagulants group

Patients in the anticoagulant group received a daily dose of 40 mg of enoxaparin within 24 hours following their surgery and continued this regimen until day 7 after transplantation, provided that the platelet count exceeded 30,000/μL, the estimated glomerular filtration rate (eGFR) was higher than 30 mL/min/1.73m^2^, and there were no major bleeding events.

In order to assess the efficacy of prophylactic anticoagulation, anti-Xa levels were measured four hours after the third dose of enoxaparin.

- 1. Control group

The control group received a daily dose of normal saline (0.9% NaCl) within 24 hours following their surgery, with this regimen continuing until day 7 after transplantation.

- 1. Postoperative Monitoring Protocol

Following transplantation, all patients were transferred to the intensive care unit (ICU). Doppler ultrasonography (DUS) was performed on a daily basis during the initial two-week postoperative period and on a weekly basis during the subsequent three-week postoperative period. The objective of this procedure was to confirm the patency of the portal vein and extremity veins.

Following surgery, routine blood tests, liver and kidney function assessments, a rapid thrombelastogram (rTEG), and monitoring of protein S (PS), protein C (PC), and anti-Xa levels were conducted.

- 1. Trial Flow Chart


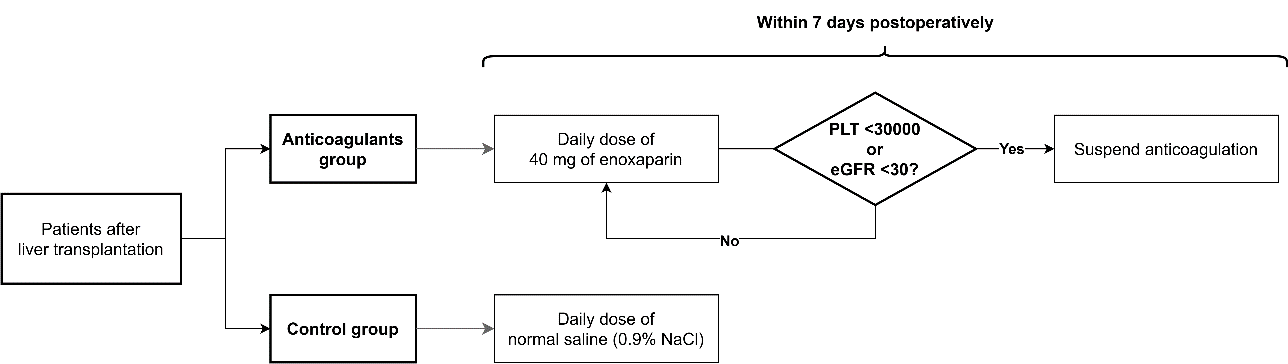


1. **Clinical Outcomes**
   1. Primary Outcome
      1. Primary efficacy outcome: Incidence of VT.

The term VT was defined as the sum of venous thrombosis, including both PVT and DVT. The definition did not include superficial vein thrombosis of the extremities that did not require intervention.

- - 1. Primary safety outcome: Incidence of major bleeding events.
  1. Secondary Outcome
     1. All-cause mortality.
     2. The incidences of PVT and DVT events.
  2. Diagnosis and Treatments of Endpoint Events
     1. Thrombosis events

The diagnosis of PVT and DVT was based on the results of daily DUS examinations and the patient's clinical presentation. A daily ultrasound examination was conducted, which included the grafts, portal veins, hepatic arteries, hepatic veins, inferior vena cava, deep and superficial veins of the extremities. This was performed by several ultrasound physicians who were not part of the research team.

All patients underwent a plain thoracic CT scan to detect any lung abnormalities. For those diagnosed with DVT via DUS, an enhanced thoracic CT scan was performed to screen for PE if their PaO₂/FiO₂ ratio (the ratio of arterial oxygen partial pressure to inspired oxygen fraction) dropped below 200, with cases of pleural effusion and pulmonary atelectasis excluded.

Once a diagnosis of PVT or DVT was established, therapeutic anticoagulation with low molecular weight heparin and rivaroxaban was initiated for a minimum of six months. Surgical portal vein thrombectomy is performed as soon as possible for portal vein embolism occurring in the early postoperative period (less than one month postoperatively), while percutaneous thrombectomy is the mainstay of PVT in the late postoperative period (greater than one month postoperatively).

- - 1. Major bleeding events

Diagnosis of major bleeding follows International Society on Thrombosis and Haemostasis (ISTH) criteria^1^:

- - - 1. Fatal bleeding, and/or;
      2. Symptomatic bleeding in a critical area or organ, such as intracranial, intraspinal, intraocular, retroperitoneal, intra-articular or pericardial, or intramuscular with compartment syndrome, and/or;
      3. Bleeding causing a fall in hemoglobin levels of 20 g/L or greater, or leading to a transfusion of 2 U or more of red blood cells (RBC).

Furthermore, the colour, character and quantity of the abdominal drainage fluid were observed, as well as the presence of clinical symptoms such as black stools, in order to assist in the diagnosis of abdominal and gastrointestinal bleeding. In the event of a suspicion of intracranial haemorrhage, a head CT scan should be performed without delay in order to confirm the diagnosis.

The treatment of bleeding is based on the administration of blood transfusions. Indications for RBC transfusion was strictly based on National Institute for Health and Care Excellent (NICE) Blood Transfusion Guideline^2^:

- - - 1. Use restrictive red blood cell transfusion thresholds for patients who need red blood cell transfusions and who do not: have major haemorrhage, or have acute coronary syndrome, or need regular blood transfusions for chronic anemia.
      2. When using a restrictive red blood cell transfusion threshold, consider a threshold of 70 g/L and a hemoglobin concentration target of 70–90 g/L after transfusion.

In accordance with the regulations of the research centers, routine blood tests must be performed to confirm compliance with transfusion indications before ICU physicians and surgeons order RBC transfusions, unless the patient's condition is so critical that there is a “major hemorrhage” as defined by the NICE guideline^2^:

- - - 1. The loss of more than 1 blood volume within 24 hours (around 70 ml/kg, or more than 5L in a 70 kg adult).
      2. A loss of 50% of total blood volume in under 3 hours.
      3. Bleeding in excess of 150 ml/min in adults.
      4. As a practical clinical definition, bleeding which leads to: a systolic blood pressure of less than 90 mmHg, or a heart rate of more than 110 beats per minute in adults.

According to NICE guideline^2^, consider single-unit RBC transfusions for adults who do not have active bleeding. After each single‑unit red blood cell transfusion, clinically reassess and check hemoglobin levels, and give further transfusions if needed. The transfusion volume was estimated according to the experience that transfusion of 1 unit of RBC raises hemoglobin by 10 g/L. With compliance with the above transfusion regulations, patients who received more than 2 units of RBCs for bleeding within 24 hours were defined as major bleeding events, regardless of whether hemoglobin decreased by more than 20 g/L.

In patients with poor coagulation, coagulation factors are supplemented. Surgical intervention should be undergone immediately if surgeons evaluate and conclude that abdominal hemorrhage cannot be controlled by blood transfusion and conservative treatment, or surgical haemorrhage is highly suspected. The surgical interventions include exploratory laparotomy and vascular repair.

- 1. Other Clinical Information
     1. Preoperative Baseline Data
        1. Demographic data

Age, gender, ethnicity, height, religion, weight, body mass index (BMI).

- - - 1. Laboratory tests

Blood routine examination, liver and kidney function, electrolytes, AFP, PIVKA-II, hepatitis markers and HBV-DNA, coagulation, inflammatory factors, Child-Pugh score and classification.

- - - 1. Surgical history

History of upper abdominal surgery, transarterial chemoembolization (TACE), portal vein embolization (PVE).

- - - 1. Comorbidities

Cardiovascular disease, cerebrovascular disease, respiratory disease, diabetes mellitus, etc.

- - - 1. Imaging

Liver enhanced MRI, whole abdomen enhanced CT, chest CT, DUS, etc.

- - 1. Intraoperative Data

Operation time, duration of cold ischemia, duration of anhepatic stage, method of vena cava reconstruction, intraoperative blood loss, intraoperative blood transfusion (including red blood cells, plasma, platelets, cold precipitation, and autologous salvaged blood transfusion), whether or not the portal vein was thrombosed, intraoperative blood pressure, etc.

- - 1. Postoperative observation data
       1. Intake and output of patients will be recorded daily.
       2. Patients were monitored daily for two weeks postoperatively for blood routine examination, liver and kidney function, electrolytes, coagulation routine, ACT, thromboelastography, and DUS.

1. **Sample Size & Randomization**

The objective of this study was to ascertain whether there was a difference in the rate of thrombosis events between the anticoagulant and control groups. Accordingly, a sample size estimation of two proportions was employed.

The study centre implemented a more rigorous thrombus screening strategy, including daily DUS screening for post-transplant patients. This approach led to the detection of a significant number of asymptomatic isolated calf muscle vein thrombosis (ICMVT) cases, as observed in our centre's experience. This suggests that diagnostic strategies based solely on clinical symptoms may underestimate the true incidence of DVT. A previous study by Annamalai A et al. reported a 15.5% incidence of DVT in patients screened daily by DUS, which is consistent with our prior experience^3^. Consequently, we postulated an 18% (P1) incidence of VT in the control group (DVT: 15%, PVT: 3%).

A multitude of studies have demonstrated that the use of enoxaparin reduces the risk of deep vein thrombosis (DVT) by more than 50% in patients undergoing major orthopedic surgery^4^. Mismetti P. et al. reported that the application of enoxaparin after major general surgery reduced the incidence of DVT by 72%^5^, however, the benefit is uncertain due to the poor quality of the current evidence^6^. A paucity of studies exists on the efficacy of LMWH in reducing PVT following liver surgery, due to the low incidence of this complication. In cirrhotic patients with Child-Pugh classes B7-C10, prophylactic anticoagulation with enoxaparin demonstrated a 68% reduction in the incidence of PVT^7^. In light of the aforementioned considerations, we postulated that enoxaparin would result in a 50% reduction in VT events (18% [P1] to 9% [P2] in the anticoagulant group). Furthermore, we assumed a 2.5% drop rate within 90 days postoperatively, given the high compliance of post-LT patients and the relatively short follow-up period for the primary outcomes. The required sample size was estimated using PASS 15 Power Analysis and Sample Size Software (NCSS, LLC., Kaysville, Utah, USA).

The sample size is calculated as follows:

| **Numeric Results for Testing Two Proportions using the Z-Test with Unpooled Variance** | | | | | | | | | | | | | | | | | |
| --- | --- | --- | --- | --- | --- | --- | --- | --- | --- | --- | --- | --- | --- | --- | --- | --- | --- |
| H0: P1 - P2 = 0. H1: P1 - P2 = D1 ≠ 0. | | | | | | | | | | | | | | | | | |
| **Target**  **Power** | | **Actual**  **Power** | | **N1** | | **N2** | | **N** | | **P1** | | **P2** | | **Diff**  **D1** | | **α** | |
| 0.80 | | 0.80108 | | 223 | | 223 | | 446 | | 0.1800 | | 0.0900 | | 0.0900 | | 0.0500 | |
| **Dropout-Inflated Sample Size** | | | | | | | | | | | | | | | | | |
| **Dropout Rate** | ─── Sample Size ─── | | | | | | Dropout-Inflated  Enrollment  ─── Sample Size ─── | | | | | | Expected Number of  ─── Dropouts ─── | | | | |
|  | **N1** | | **N2** | | **N** | | **N1'** | | **N2'** | | **N'** | | **D1** | | **D2** | | **D** |
| 2.5% | 223 | | 223 | | 446 | | 229 | | 229 | | 458 | | 6 | | 6 | | 12 |

Patients were randomly allocated (1:1) to either the anticoagulant group or the control group using stratified randomization, with the allocation stratified according to six different surgeons. The randomization was created using IBM SPSS Statistics 26.0, and the computer-generated list of random number was used for group allocation, with subject numbers placed in separate, unmarked envelopes. The investigator then proceeded to open the envelopes for the subjects, thereby identifying the subject groups.

1. **Statistical Analysis**
   1. Descriptive analysis

For the sample description, demographic and preoperative/intraoperative clinical data for the two groups were reported. The Shapiro-Wilk test was used to evaluate the normality of variable distributions. Continuous data following a normal distribution were presented as the mean ± standard deviation, whereas non-normally distributed data were reported as the median and interquartile range (IQR). Dichotomous data were expressed as counts and percentages; specifically, sex is reported in the form of male/female ratio.

- 1. Efficacy and safety analysis

Both ITT and PP analyses were conducted for all outcomes. All allocated patients were included in the ITT dataset, regardless of compliance. In contrast, the PP dataset included only those who received the intervention according to protocol, excluding patients who met the PP analysis exclusion criteria, such as those who did not receive prophylactic anticoagulation (including cases where endpoint events occurred prior to the first enoxaparin dose). Additionally, patients who required RRT within the first 7 days post-LT were excluded, as they needed anticoagulation with unfractionated heparin or fondaparinux during therapy.

The incidence and risk ratios (RRs) for the primary and secondary outcomes were calculated and compared between the two groups by χ^2^ test using cross-tabulation function of IBM SPSS Statistics 26.0 (IBM corp., Armonk, NY, USA). The formulas of RRs and their 95% CI are as follows:

$$RR=\frac{a/{(a+b)}}{c/{(c+d)}} 95\% CI\in(e^{[\ln\left( RR \right)-1.96\sqrt{\frac{1}{a}-\frac{1}{a+b}+\frac{1}{c}-\frac{1}{c+d}}]},e^{[\ln\left( RR \right)+1.96\sqrt{\frac{1}{a}-\frac{1}{a+b}+\frac{1}{c}-\frac{1}{c+d}}]})$$

*a:* number of events in the intervention group; *b:* number of non-events in the intervention group;*c:* number of events in the control group; *d:* number of non-events in the control group

Cumulative incidence or survival probabilities were calculated for primary and secondary outcomes.HRs and their 95% CIs were also estimated. Detailed statistical methods are described below:

- - 1. Thrombosis (venous thrombosis, PVT and DVT) and major bleeding events

For competing risk analyses, thrombosis, major bleeding and all-cause death events were treated as competing events relative to each other. Cumulative incidence functions (CIF) were calculated for both outcomes, with CIF curves generated accordingly and comparisons assessed using Gray’s test^8^. Fine-Gray subdistribution hazard models were used to calculate HRs for those events, adjusting for potential confounding variables. Given that our thrombus screening strategy identified a large number of asymptomatic ICMVT cases, which differs from previous reports, the CIF curves and HRs for both ICMVT and proximal DVT (PDVT) were also reported. All CIF curves and HRs were generated with 95% CIs.

For the Fine-Gray subdistribution hazard models, potential confounding variables were adjusted informed by clinical practice experience as follows:

- - - 1. For venous thrombosis events, HCC, preoperative PVT, and surgeons (stratification factor) were adjusted. Malignancy is one of the major risk factors for DVT^9^, and since HCC was prevalent in nearly 50% of the LT patient population at the study centres, it was used as an adjustment factor for estimating adjusted HRs for VT and DVT events. Preoperative PVT is a known risk factor for PVT after LT^10^, and as such, it was used as an adjustment factor when estimating HRs for VT and PVT.

Although the surgeons in this study were experienced in LT, variations in anastomotic techniques and other surgical practices may influence postoperative complications, especially PVT, HAT, and major bleeding events, as well as the overall prognosis. Hence, the surgeon was included both as a randomisation stratification factor and an adjustment factor when estimating HRs for VT, PVT, and major bleeding events.

- - - 1. For major bleeding events, surgeons and intraoperative blood loss were adjusted. Risk factors for major bleeding after LT are not yet clearly defined. Jung J et al.^11^ reported that increased intraoperative blood loss is a major risk factor for postoperative bleeding after LT, which aligns with the clinical experience at our study centre. Increased intraoperative blood loss can be linked to poor liver function and coagulation status in the patient, and may also result in excessive consumption of coagulation factors, thus elevating the risk of major bleeding post-LT. Therefore, intraoperative blood loss was used as an adjustment factor for estimating HRs for major bleeding events. The rationale for adjusting for surgeons in the estimation of HRs for major bleeding events was previously described.
    1. Overall Survival

The Kaplan-Meier method was used to estimate survival probabilities, with log-rank p-values for comparisons. A Cox proportional hazards model was applied to calculate HRs for survival, adjusting for potential confounding variables. Specifically, survival curves and HRs for perioperative overall survival were also generated. All CIF curves and HRs were generated with 95% CIs.

For the Cox proportional hazards model, potential confounding variables including operation time and surgeons were adjusted. Numerous prognostic factors influence outcomes in patients after LT. Prior studies^12, 13^ identified prolonged operation time and diabetes as major factors affecting patient prognosis, aligning with the clinical experience at our study centre. Consequently, they were incorporated as an adjustment factor when estimating HRs for survival. The rationale for adjusting for surgeons in the estimation of HRs for major bleeding events was previously described.

Missing data were imputed using the median values (applies to operation time and intraoperative blood loss). P-values were calculated as two-sided, with statistical significance defined as P <0.05. All analyses described above were conducted using R version 4.4.0 (R Foundation for Statistical Computing, Vienna, Austria).

- 1. Subgroup analysis

Potential interest subgroups will be analysed with an aim of exploration. The following criteria will be used to group the subgroups: HCC, MELD score (≥30), high risk of postoperative bleeding, preoperative PVT and other risk factors for postoperative PVT. Patients with intraoperative blood loss exceeding 2000 mL or a platelet count lower than 30,000/μL for more than four days within seven days post-operation are considered to be at high risk of postoperative bleeding. Patients with a history of pre-operative variceal bleeding, pre-operative PVT, portal vein thrombectomy, or non-physiological reconstruction of portal vein inflow were deemed as at high risk of post-operative PVT according to the literature^10^.

Similarly, A Cox proportional hazards model or Fine-Gray subdistribution hazard models was applied to calculate HR for survival, with 95% CIs, adjusting for surgeons (randomisation stratification factor). It should be noted that subgroup analyses are exploratory in nature; their results may lack robustness and should not be considered as primary conclusions.

1. **Ethics & Others**
   1. Research Ethics Approval

The subjects were obtained from inpatients of West China Hospital of Sichuan University and the First Affiliated Hospital of Chongqing Medical University. Prior to commencing the trial, approval will be sought from the Ethics Committee on Biomedical Research, West China Hospital of Sichuan University. The trial is conducted in accordance with the Declaration of Helsinki and is subject to periodic review by the Ethics Committee on Biomedical Research, West China Hospital of Sichuan University, and the Ethics Committee of the First Affiliated Hospital of Chongqing Medical University. Following the provision of informed consent, all subjects will be enrolled in the study.

- 1. Confidentiality

The researcher guarantees that the subjects' personal data will be safeguarded and will not be utilized for statistical analysis or disclosed in any manner. The subjects' medical records are stored and safeguarded within the hospital information system, which is rigorously managed in accordance with the pertinent regulations of West China Hospital of Sichuan University and the First Affiliated Hospital of Chongqing Medical University. Only the investigator and physicians will have access to this information.

- 1. Perioperative Management & Post-trial Care

The surgical procedures and anticoagulation regimens employed in this study were all well-established techniques that are routinely used in clinical practice. They did not involve the use of other unconventional new drugs or techniques. Consequently, the surgical and postoperative management of the patients was conducted in accordance with the standard procedures, and the post-transplantation patients were monitored in accordance with the standard protocols.

The position of surgeon-in-chief is to be filled by a senior physician with more than 10 years of experience in liver surgery and more than 30 liver transplants per year over the past three years.

Maintenance immunosuppression was achieved using tacrolimus, mycophenolate, and prednisone. Calcineurin inhibitor therapy was initiated on postoperative day 1 or 2, while standard recipient variables and patient history were recorded.

- 1. Observation & Record of Adverse Events
     1. Definition of Adverse Event

An adverse medical event that occurs subsequent to the administration of a pharmaceutical agent or therapeutic intervention to a patient or clinical trial subject, and which is not necessarily causally related to the treatment in question.

- - 1. Serious adverse events

Events requiring hospitalization, prolonged hospitalization, disability, affecting work ability, life-threatening or death, causing congenital malformations, etc. occurring in the course of a clinical trial.

- - 1. Record and report of adverse events

It is possible that adverse events may occur during the course of treatment. Once an adverse event (including important adverse events) occurs, it is important to record the occurrence of adverse events in detail on the case report form. This should include information on the clinical manifestations, treatment and duration, regression, and the relationship with the drug; laboratory test abnormalities. It is also necessary to monitor the patient until the results of the test return to normal or to the level of the pre-drug, or to determine that it is not related to the trial drug. In the event of a serious adverse event, the relevant parties must be informed within 24 hours. This includes completion of the serious adverse event form and reporting to the sponsor, the ethics committee, the CFDA Safety Supervision Department and the health administration department.

1. **Changes to Protocol after Trial Commencement**
   1. Number of Arms

The study was initially registered with three groups: the control group, the fixed group, and the adjusted group. However, the COVID-19 pandemic significantly disrupted the availability of anti-Xa level monitoring during patient recruitment. Consequently, the anti-Xa-based enoxaparin regimen planned for the adjusted group could not be implemented, leading to the termination of this group. No patients were recruited into the adjusted group, resulting in a modification of the study design to two groups: the anticoagulant group and the control group, as described above.

- 1. Sample Size and Outcomes

The sample size initially reported in the registry entry was based on a previous study examining the effects of prophylactic enoxaparin on the prevention of PVT after hepatectomy, which reported an 80% decrease (10% to 2%) in the incidence of PVT^9^. The estimated incidence of HAT was 5%^14, 15^. It was initially hypothesised that the incidence of vascular thrombosis events in the control group would be 15% (10% PVT and 5% HAT). Based on prior studies, it was further hypothesised that enoxaparin would reduce venous thrombosis events by 80% (from 15% to 3% in the anticoagulant group). A dropout rate of 2.5% within 90 days postoperatively was assumed, leading to an estimated sample size of 99 participants per group across the three groups.

However, at the start of the study, several issues emerged: (a) the adjusted group was terminated, (b) we realized that enoxaparin, theoretically, has no preventive effect on HAT and is more likely to prevent DVT, and (c) the incidence of PVT was overestimated. Consequently, the sample size was re-estimated in December 2020, and the primary outcome was changed to VT.

The protocol amendments were reviewed and approved by the ethics committee, and subsequently registered with the Chinese Clinical Trial Registry. To minimise bias, the results for the original outcome are reported in the Supplemental Results section.

Supplementary Results

1. **Report of Revised Outcomes**

After the study commenced, investigators observed that the incidence of PVT events was far lower than expected, resulting in a significantly underpowered original sample size. Given the practical constraints on participant recruitment, the primary outcome was revised to venous thrombosis events, defined as the combined incidence of PVT and DVT in December 2020. Simultaneously, HAT was removed as a primary outcome, as low-molecular-weight heparin theoretically has no preventive effect on arterial thrombosis.

To minimise bias, the results for the original outcome are reported in the Supplemental Results.

- 1. HAT

HAT events occured in 2 patients (0.9%) in the anticoagulant group, and none in the control group within first 90 days post-LT (*P* = 0.479). The RR could not be estimated as there were no events in the control group. Additionally, the HR was not estimated due to the small number of events, and no analysis was conducted using a competing risk model.

- 1. Laboratory indicators within 7 days postoperatively

No significant differences were observed in median laboratory indicators, including rTEG parameters (reaction time [R], kinetics time [K], maximum amplitude [MA], α-angle [α], and clot strength [G]), protein C, protein S, and antithrombin, within seven days postoperatively between the two groups (Figure S4).

1. **Report of Unadjusted HRs of Primary and Secondary Outcomes**

To ensure the robustness of the models and minimise false-positive results, unadjusted HRs in the ITT population were also reported (Table S1). HRs for primary and secondary outcomes remained consistent before and after covariate adjustment. Notably, while HRs for ICMVT were statistically significant after adjustment, they only exhibited a trend towards significance before adjustment; thus, this finding should be interpreted with caution.

Supplementary Figures and Tables

**Figure S1** Monitoring results of Anti-Xa concentration after the third dose of enoxaparin


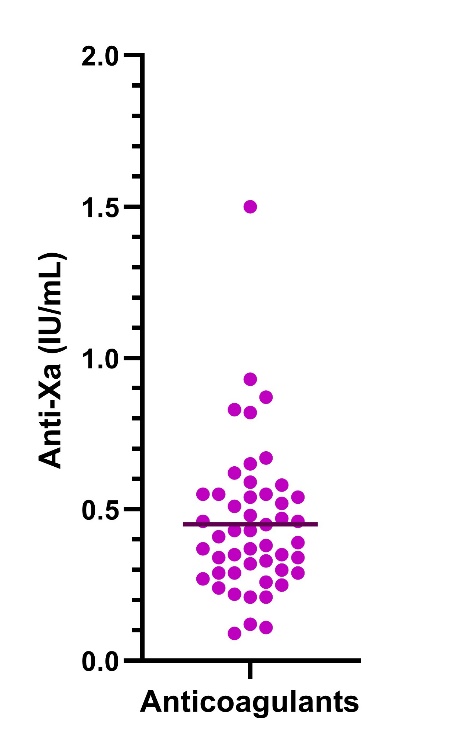


**Figure S2** Competing Risk and Survival Probability of Secondary Outcomes

(A) Competing risk of postoperative PVT event.

(B) Competing risk of postoperative DVT event.

(C) Competing risk of postoperative ICMVT event.

(D) Competing risk of postoperative PDVT event.

(E) Overall survival.

(F) Overall survival in perioperative period.


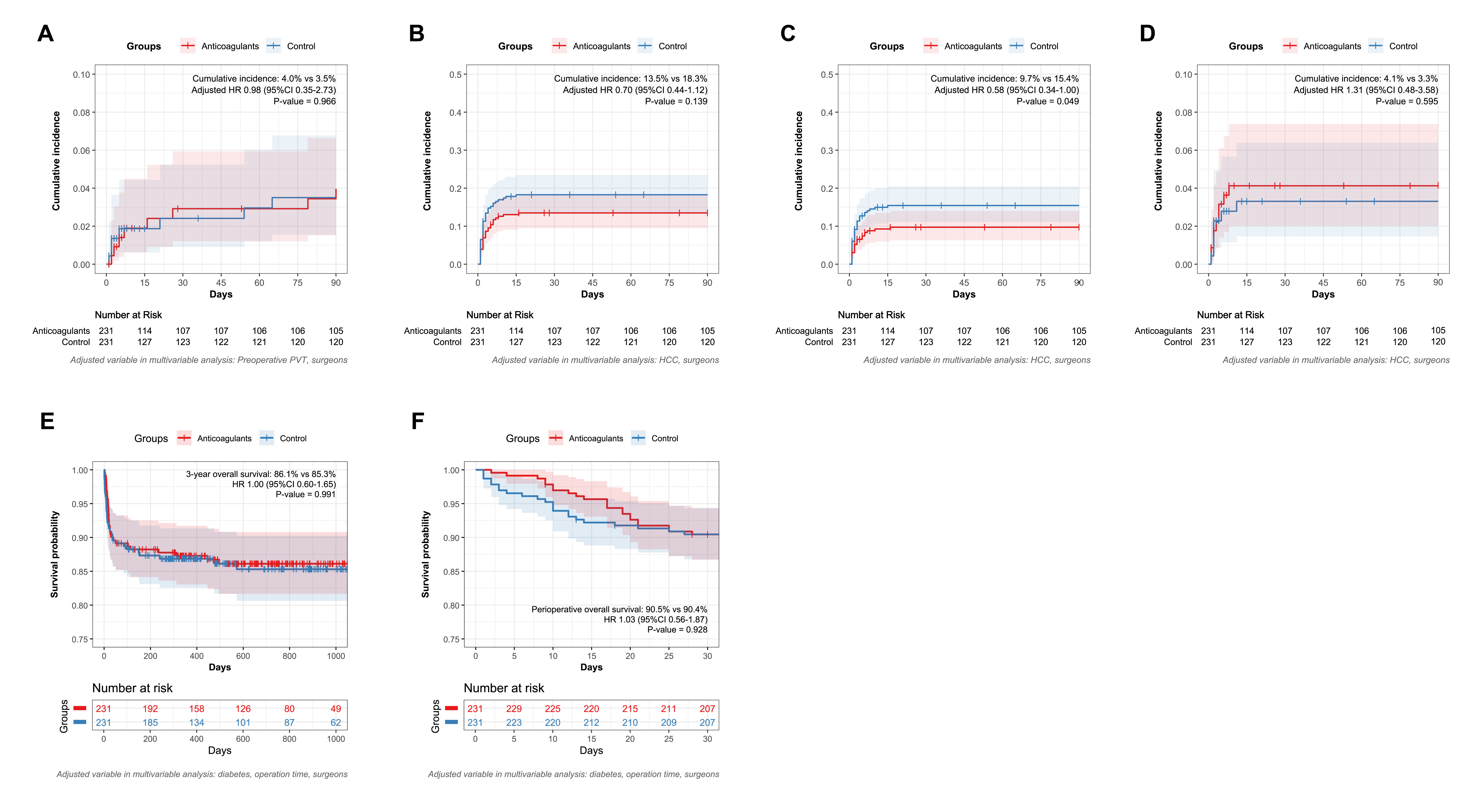


**Figure S3** Exploratory Subgroup Analysis: Adjusted HRs of Secondary Outcomes in Subgroups

(A) HRs of postoperative PVT event in subgroups.

(B) HRs of postoperative DVT event in subgroups.

(C) HRs of postoperative overall survival in subgroups.


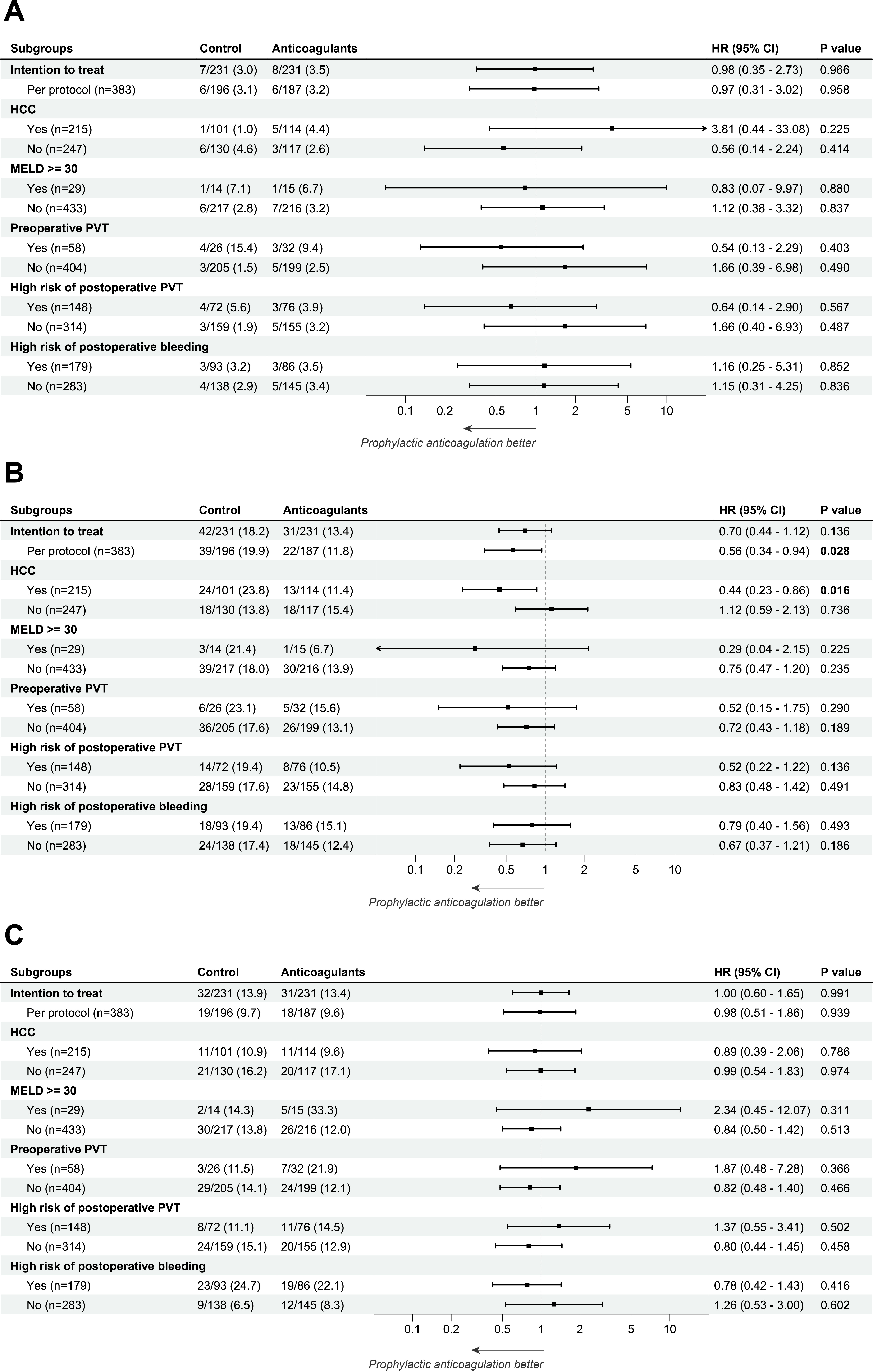


**Figure S4** Laboratory indicators within 7 days postoperatively


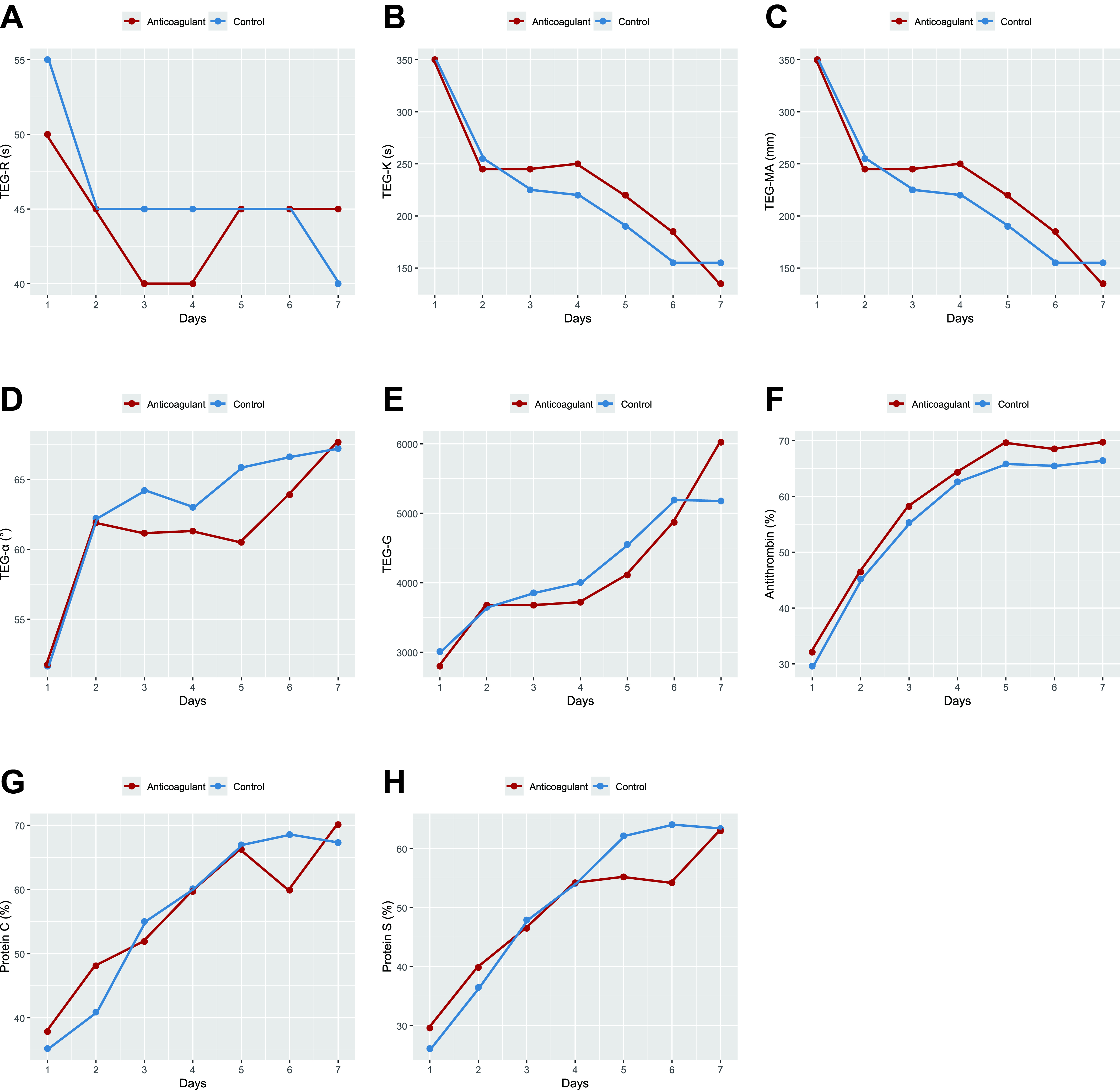


**Table S1** Adjusted HRs and unadjusted HRs of primary and secondary outcomes in ITT population

| **Outcomes** | **Adjusted HR** | **P value** | **Unadjusted HR** | **P value** |
| --- | --- | --- | --- | --- |
| Venous thrombosis | 0.76 (0.50-1.16) | 0.198 | 0.79 (0.52-1.19) | 0.255 |
| PVT | 0.98 (0.35-2.73) | 0.966 | 1.10 (0.40-3.01) | 0.856 |
| DVT | 0.70 (0.44-1.12) | 0.139 | 0.72 (0.45-1.13) | 0.152 |
| *ICMVT* | 0.58 (0.34-1.00) | **0.049** | 0.61 (0.36-1.03) | 0.064 |
| *PDVT* | 1.31 (0.48-3.58) | 0.595 | 1.25 (0.47-3.34) | 0.657 |
| All-cause mortality | 1.00 (0.60-1.65) | 0.991 | 0.94 (0.57-1.53) | 0.794 |
| *Perioperative all-cause mortality* | 1.03 (0.56-1.87) | 0.928 | 0.98 (0.54-1.77) | 0.941 |
| Major bleeding | 1.40 (1.00-1.94) | **0.047** | 1.43 (1.03-1.99) | **0.032** |

**Table S2** Summary of causes of death, categorized according to the postoperative period in ITT population

| **Time to death** | **Cause of death** | **Anticoagulants group** | **Control group** | **Total** |
| --- | --- | --- | --- | --- |
| ≤30 days  *(perioperative period)* | Infection | 14 | 13 | 27 |
|  | Major bleeding | 1 | 3 | 4 |
|  | PVT or HAT | 2 | 2 | 4 |
|  | Graft failure | 2 | 2 | 4 |
|  | Stroke | 2 | 0 | 2 |
|  | Others | 1 | 2 | 3 |
|  | **Total** | 22 | 22 | 44 |
|  | **30-day OS** | **90.4%** | **90.5%** | **90.4%** |
| 30-90 days | Infection | 2 | 3 | 5 |
|  | PVT or HAT | 1 | 0 | 1 |
|  | Graft failure | 0 | 1 | 1 |
|  | **Total** | 3 | 4 | 7 |
|  | **90-day OS** | **89.1%** | **88.7%** | **88.9%** |
| 90-365 days | Infection | 2 | 3 | 5 |
|  | Graft failure | 1 | 0 | 1 |
|  | Tumour recurrence | 0 | 1 | 1 |
|  | Others | 1 | 0 | 1 |
|  | **Total** | 4 | 4 | 8 |
|  | **1-year OS** | **87.3%** | **86.9%** | **87.0%** |
| >365 days | Infection | 2 | 1 | 3 |
|  | Tumour recurrence | 0 | 1 | 1 |
|  | **Total** | 2 | 2 | 4 |
|  | **3-year OS** | **86.1%** | **85.3%** | **85.7%** |
| All times | Infection | 20 | 20 | 40 |
|  | Graft failure | 3 | 3 | 6 |
|  | PVT or HAT | 3 | 2 | 5 |
|  | Major bleeding | 1 | 3 | 4 |
|  | Stroke | 2 | 0 | 2 |
|  | Tumour recurrence | 0 | 2 | 2 |
|  | Others | 2 | 2 | 4 |
|  | **Total** | 31 | 32 | 63 |

References

1. Schulman S, Kearon C. Definition of major bleeding in clinical investigations of antihemostatic medicinal products in non-surgical patients. *J Thromb Haemost* 2005;**3**(4): 692-694.

2. National Clinical Guideline Centre. National Institute for Health and Care Excellence: Guidelines. In: *Blood Transfusion*. National Institute for Health and Care Excellence (NICE): London, 2015.

3. Annamalai A, Kim I, Sundaram V, Klein A. Incidence and risk factors of deep vein thrombosis after liver transplantation. *Transplant Proc* 2014;**46**(10): 3564-3569.

4. Falck-Ytter Y, Francis CW, Johanson NA, Curley C, Dahl OE, Schulman S, Ortel TL, Pauker SG, Colwell CW, Jr. Prevention of VTE in orthopedic surgery patients: Antithrombotic Therapy and Prevention of Thrombosis, 9th ed: American College of Chest Physicians Evidence-Based Clinical Practice Guidelines. *Chest* 2012;**141**(2 Suppl): e278S-e325S.

5. Mismetti P, Laporte S, Darmon JY, Buchmüller A, Decousus H. Meta-analysis of low molecular weight heparin in the prevention of venous thromboembolism in general surgery. *Br J Surg* 2001;**88**(7): 913-930.

6. Anderson DR, Morgano GP, Bennett C, Dentali F, Francis CW, Garcia DA, Kahn SR, Rahman M, Rajasekhar A, Rogers FB, Smythe MA, Tikkinen KAO, Yates AJ, Baldeh T, Balduzzi S, Brożek JL, Ikobaltzeta IE, Johal H, Neumann I, Wiercioch W, Yepes-Nuñez JJ, Schünemann HJ, Dahm P. American Society of Hematology 2019 guidelines for management of venous thromboembolism: prevention of venous thromboembolism in surgical hospitalized patients. *Blood Adv* 2019;**3**(23): 3898-3944.

7. Villa E, Cammà C, Marietta M, Luongo M, Critelli R, Colopi S, Tata C, Zecchini R, Gitto S, Petta S, Lei B, Bernabucci V, Vukotic R, De Maria N, Schepis F, Karampatou A, Caporali C, Simoni L, Del Buono M, Zambotto B, Turola E, Fornaciari G, Schianchi S, Ferrari A, Valla D. Enoxaparin prevents portal vein thrombosis and liver decompensation in patients with advanced cirrhosis. *Gastroenterology* 2012;**143**(5): 1253-1260.e1254.

8. Gray RJ. A Class of K-Sample Tests for Comparing the Cumulative Incidence of a Competing Risk. *The Annals of Statistics* 1988;**16**(3): 1141-1154.

9. Godier A, Lasne D, Pernod G, Blais N, Bonhomme F, Bounes F, Bourguignon A, Cohen A, de Maistre E, Fontana P, Galanaud JP, Huet DG, Godon A, Gouin-Thibault I, Jebara S, Laporte S, Lecompte T, Longrois D, J HL, Le Gal G, Gruel Y, Mansour A, Martin AC, Mazighi M, Morange PE, Motte S, Mullier F, Nguyen P, Rosencher N, Roullet S, Roy PM, Schved JF, Sevestre MA, Sié P, Susen S, Tacquard C, Vincentelli A, Zufferey P, Mismetti P, Albaladejo P. Prevention of perioperative venous thromboembolism: 2024 guidelines from the French Working Group on Perioperative Haemostasis (GIHP) developed in collaboration with the French Society of Anaesthesia and Intensive Care Medicine (SFAR), the French Society of Thrombosis and Haemostasis (SFTH) and the French Society of Vascular Medicine (SFMV) and endorsed by the French Society of Digestive Surgery (SFCD), the French Society of Pharmacology and Therapeutics (SFPT) and INNOVTE (Investigation Network On Venous ThromboEmbolism) network. *Anaesth Crit Care Pain Med* 2024: 101446.

10. Montalvá E, Rodríguez-Perálvarez M, Blasi A, Bonanad S, Gavín O, Hierro L, Lladó L, Llop E, Pozo-Laderas JC, Colmenero J. Consensus Statement on Hemostatic Management, Anticoagulation, and Antiplatelet Therapy in Liver Transplantation. *Transplantation* 2022;**106**(6): 1123-1131.

11. Jung JW, Hwang S, Namgoong JM, Yoon SY, Park CS, Park YH, Lee HJ, Park HW, Park GC, Jung DH, Song GW, Ha TY, Ahn CS, Kim KH, Moon DB, Ko GY, Sung KB, Lee SG. Incidence and Management of Postoperative Abdominal Bleeding After Liver Transplantation. *Transplantation Proceedings* 2012;**44**(3): 765-768.

12. Filali Bouami S, Gwiasda J, Beneke J, Kaltenborn A, Liersch S, Suero EM, Koch HF, Krauth C, Klempnauer J, Schrem H. Prognostic factors for long-term survival after adult liver transplantation. *Langenbecks Arch Surg* 2018;**403**(4): 495-508.

13. Asrani SK, Saracino G, O'Leary JG, Gonzalez S, Kim PT, McKenna GJ, Klintmalm G, Trotter J. Recipient characteristics and morbidity and mortality after liver transplantation. *J Hepatol* 2018;**69**(1): 43-50.

14. Bekker J, Ploem S, de Jong KP. Early hepatic artery thrombosis after liver transplantation: a systematic review of the incidence, outcome and risk factors. *Am J Transplant* 2009;**9**(4): 746-757.

15. Duffy JP, Hong JC, Farmer DG, Ghobrial RM, Yersiz H, Hiatt JR, Busuttil RW. Vascular complications of orthotopic liver transplantation: experience in more than 4,200 patients. *J Am Coll Surg* 2009;**208**(5): 896-903; discussion 903-895.
